# Microbial Warfare on Three Fronts: Mixed Biofilm of *Aspergillus fumigatus* and *Staphylococcus aureus* on Primary Cultures of Human Limbo-Corneal Fibroblasts

**DOI:** 10.3389/fcimb.2021.646054

**Published:** 2021-08-16

**Authors:** Adrián Ramírez-Granillo, Luis Antonio Bautista-Hernández, Víctor Manuel Bautista-De Lucío, Fátima Sofía Magaña-Guerrero, Alfredo Domínguez-López, Itzel Margarita Córdova-Alcántara, Néstor O. Pérez, María de los Angeles Martínez-Rivera, Aída Verónica Rodríguez-Tovar

**Affiliations:** ^1^Medical Mycology Laboratory, National School of Biological Sciences (ENCB)-Instituto Politécnico Nacional (IPN), Department of Microbiology, Mexico City, Mexico; ^2^Ocular Microbiology and Proteomics Laboratory, Research Unit, “Conde de Valenciana Private Assistance Foundation”, Mexico City, Mexico; ^3^Cell Biology and Amniotic Membrane Laboratory, Research Unit, “Conde de Valenciana Private Assistance Foundation”, Mexico City, Mexico; ^4^Research and Development Department Probiomed SA de CV, Tenancingo Edo de Mex, Mexico

**Keywords:** *Aspergillus fumigatus*, *Staphylococcus aureus*, fungal–bacterial interaction, mixed fungal–bacterial biofilms, human limbo-corneal fibroblast cells

## Abstract

**Background:**

Coinfections with fungi and bacteria in ocular pathologies are increasing at an alarming rate. Two of the main etiologic agents of infections on the corneal surface, such as *Aspergillus fumigatus* and *Staphylococcus aureus*, can form a biofilm. However, mixed fungal–bacterial biofilms are rarely reported in ocular infections. The implementation of cell cultures as a study model related to biofilm microbial keratitis will allow understanding the pathogenesis in the cornea. The cornea maintains a pathogen-free ocular surface in which human limbo-corneal fibroblast cells are part of its cell regeneration process. There are no reports of biofilm formation assays on limbo-corneal fibroblasts, as well as their behavior with a polymicrobial infection.

**Objective:**

To determine the capacity of biofilm formation during this fungal–bacterial interaction on primary limbo-corneal fibroblast monolayers.

**Results:**

The biofilm on the limbo-corneal fibroblast culture was analyzed by assessing biomass production and determining metabolic activity. Furthermore, the mixed biofilm effect on this cell culture was observed with several microscopy techniques. The single and mixed biofilm was higher on the limbo-corneal fibroblast monolayer than on abiotic surfaces. The *A. fumigatus* biofilm on the human limbo-corneal fibroblast culture showed a considerable decrease compared to the *S. aureus* biofilm on the limbo-corneal fibroblast monolayer. Moreover, the mixed biofilm had a lower density than that of the single biofilm. Antibiosis between *A. fumigatus* and *S. aureus* persisted during the challenge to limbo-corneal fibroblasts, but it seems that the fungus was more effectively inhibited.

**Conclusion:**

This is the first report of mixed fungal–bacterial biofilm production and morphological characterization on the limbo-corneal fibroblast monolayer. Three antibiosis behaviors were observed between fungi, bacteria, and limbo-corneal fibroblasts. The mycophagy effect over *A. fumigatus* by *S. aureus* was exacerbated on the limbo-corneal fibroblast monolayer. During fungal–bacterial interactions, it appears that limbo-corneal fibroblasts showed some phagocytic activity, demonstrating tripartite relationships during coinfection.

## Introduction

Biofilms are microbial consortiums of sessile cells fused inside an extracellular matrix (ECM) that is composed of self-excreting biomolecules by the different microbial species. Thus, biofilms are considered a link between microorganisms and the site they are trying to colonize (Fanning and Mitchell, 2012; Ramírez Granillo et al., 2015). As such, biofilms are also considered a virulence factor influencing the pathogenesis of microbial diseases (Archer et al., 2011; Gibbons et al., 2012; Peters et al., 2012). The study of polymicrobial biofilms has gained increased attention in the last few years and has focused on the study of virulence factors such as adhesion, production, and secretion of enzymes, proteins, and toxins (Karkowska-Kuleta et al., 2009; Archer et al., 2011; Gabrilska and Rumbaugh, 2015).

Fungal–bacterial interactions (FBIs) are an example of the link that exists between these microorganisms during biofilm formation over both biotic and abiotic surfaces (Frey-Klett et al., 2011; Tarkka and Deveau, 2016). Mixed fungal–bacterial biofilms (MFBBs) tend to be more prevalent than previously thought, especially in humans, and have been associated with antimicrobial resistance, postsurgical infections, and immunodeficiency diseases (Elder et al., 1996; Nucci and Marr, 2005; Wargo and Hogan, 2006; Jabra-Rizk, 2011; Peters et al., 2012; Diaz et al., 2014; Arvanitis and Mylonakis, 2015). These risk factors are determinants of the development of MFBB infections in the eye. Structurally, the ocular surface is composed of the cornea, conjunctiva, and sclera; its main function is to protect the physical integrity of the eye (Busquet and Gabarel, 2008; Jiménez-Martínez et al., 2016; Lu and Liu, 2016). This protecting process depends on its ability to regenerate the epithelial layer under the influence of human limbo-corneal fibroblast cells (HLFCs).

On the other hand, when the ocular surface is altered, changes in the microbiota can be promoted, causing ophthalmological pathologies associated with either the microbiota located in the tissue adjacent to the cornea or the conjunctiva (paucibacterial) or the resident microbiota of high pathogenic potential (pathobionts) (Chow et al., 2011; Doan et al., 2016). The microbiome of the ocular surface is mainly composed of Gram-positive bacteria such as *Propionibacterium* sp., *Corynebacterium* sp., *Staphylococcus* sp., and Gram-negative bacteria such as *Pseudomonas* sp., *Acinetobacter* sp., among others (Dong et al., 2012; Kugadas and Gadjeva, 2016; Lu and Liu, 2016).

Bacterial keratitis is the most frequent type of infectious keratitis, mostly associated with the use of contact lenses (Delgado et al., 2008; Álvarez-Félix et al., 2010; Eltis, 2011; Bispo et al., 2015). Worldwide, the main etiologic agents of bacterial keratitis are Gram-positive cocci (*Staphylococcus aureus, S. epidermidis, Streptococcus pneumoniae, S. pyogenes, S. viridans*), followed by Gram-negative bacillus *Pseudomonas aeruginosa* (Álvarez-Félix et al., 2010; Hernández-Camarena et al., 2012; Hernandez-Camarena et al., 2015; Di Zazzo et al., 2020; Dohse et al., 2020; Singh et al., 2020). Eye fungal infections or keratomycosis in the cornea are commonly produced by corneal trauma. The frequency in the isolation of specific etiologic agents is usually related to the geographic origin as well as with the climate conditions that allow the proliferation of certain fungal species. *Fusarium* sp. and *Aspergillus* sp. are the most isolated filamentous fungi (Seal and Pleyer, 2007; Vanzzini-Zago et al., 2010; Hernández-Camarena et al., 2012; Di Zazzo et al., 2020; Dohse et al., 2020; Singh et al., 2020).

A handful of retrospective studies around the world have assessed the finding of FBI during ocular surface infections (Delgado et al., 2008; Mejia-Lopez et al., 2010). Erroneous sampling, low microbial populations on the ocular surface, and the presence of non-culturable microorganisms in ocular samples have been associated with underestimation of FBIs during ocular keratitis (Kugadas and Gadjeva, 2016; Lu and Liu, 2016). Clinical manifestations of ocular disease related to MFBBs are even harder to characterize (Samimi et al., 2013).

The *in vitro* formation of polymicrobial biofilms of *Aspergillus fumigatus* (AF)–*S. aureus (SA)* isolated from patients with infectious keratitis has been demonstrated and showed an antagonistic behavior (Ramírez Granillo et al., 2015). To our knowledge, this is the first study where the formation of polymicrobial biofilms (bacteria–fungi–cells) has been assessed using primary cultures.

The aim of this study was to demonstrate the formation of mixed biofilms *in vitro* using primary cultures of HLFCs coinfected with the two main etiologic agents of infectious keratitis, *A. fumigatus* and *S. aureus*, as well as to study the effect between these microbial agents on primary cultures of human limbo-corneal fibroblasts.

## Materials and Methods

### Strains

Clinical isolates of *A. fumigatus* (Ramírez Granillo et al., 2015; González-Ramírez et al., 2016) and *S. aureus* (Ramírez Granillo et al., 2015) were kindly donated by the Instituto de Oftalmología Fundación Conde de Valenciana (IOFCV). The characterization of both isolates was carried out in the IOFCV, and the identity of the isolates was corroborated as previously reported by Ramírez-Granillo et al. (2015). *A. fumigatus* was cultured in Potato Dextrose Agar (PDA) (MCD Lab, Tlalnepantla Estado de México, México) and incubated for 5 days at 37°C. The conidia from the *A. fumigatus* culture were harvested by flooding the plate with phosphate buffer saline (PBS) added with 10% v/v Tween 20. The surface of the fungal culture was scraped with a sterile glass scraper, followed by the obtention of the microconidia suspension with a sterile pipette. Afterward, the conidia were filtered through two sterile nylon filters (44 and 37 µm) as previously reported (Mowat et al., 2010; Ramírez Granillo et al., 2015). The conidial suspension should be used immediately after extraction for the infectivity test. To avoid rapid germination of the conidia, we use an ice bath during handling; it is possible to keep *A. fumigatus* conidia for 10 h without loss of viability. The *S. aureus* strain was seeded in BHI broth (MCD Lab, Tlalnepantla Estado de México, México) and incubated overnight (ON) at 37°C under agitation. From this original culture, a stock suspension was prepared using RPMI 1640 medium supplemented with fetal bovine serum (FBS) 10% v/v heat inactivated (Gibco, Waltham, MA, USA) adjusted with the McFarland nephelometer (tube 0.5).

The isolates used in this work can be shared with the scientific community upon request.

### Primary Human Limbo-Corneal Fibroblast Cultures

Primary HLFCs were kindly donated by the IOFVC and obtained as previously reported by Luna-Baca et al. (2007). The primary cultures were used between the third and fifth passages to avoid the proliferation of particular clones. The culture of HLFCs was standardized to adapt the cells to the biofilm formation conditions. For the propagation of HLFCs, frozen vials of fibroblasts were thawed and seeded in T75 culture flasks (Sarstedt AG, Nümbrecht, Germany) using Dulbecco’s Modified Eagle Medium/Nutrient Mixture F-12 Ham (DMEM/F-12) (Sigma-Aldrich Chemical Co., St. Louis, MO, USA) supplemented with FBS 10% v/v heat inactivated. The cells were incubated at 37°C in a 5% CO_2_ atmosphere. For the microbial challenge, confluent cell monolayers were detached with a solution of 0.5% trypsin in PBS, and cell viability was assessed using trypan blue 0.1%. Viable cells were counted under a Neubauer chamber and the cellular suspension was adjusted to the standardized infection cell multiplicity index (MOI = 1; which is proportional to 50,000 HLFCs/50,000 conidia or bacteria per well) (Luna-Baca et al., 2007; Castañeda-Sanchez et al., 2013). The counted fibroblasts were seeded on flat-bottom polystyrene multi-well plates with RPMI 1640 medium supplemented with FBS 10% v/v until reaching an adequate cell confluence (80%–90% fibroblasts). The volume is relative to the type of polystyrene plate used (96-well plate, the final volume was 200 µl; 12-well plate, the final volume was 3 ml; six-well plate, the final volume was 4 ml), and the cultures were incubated under the conditions previously indicated.

### Flow Cytometry for Immune Typification of Primary Human Limbo-Corneal Fibroblast Cell Cultures

To demonstrate the phenotype of the HLFCs, the cells were stained with three monoclonal antibodies for flow cytometry analysis. Briefly, an anti-vimentin antibody (ab92547; Abcam, United Kingdom) and an anti-pan-cytokeratin antibody (CTK) (ab7753; Abcam, United Kingdom) were used as primary antibodies. Additionally, a third antibody directed against alpha smooth muscle actin (SMA) was used to stain limbo-corneal cells (ab5694; Abcam, United Kingdom). For staining, the culture was washed twice with PBS, and the cells were detached using 0.1% trypsin in PBS and suspended in DMEM-F12 supplemented with FBS 10% v/v. The cells were fixed and permeabilized with the BD Cytofix/Cytoperm™ Fixation/Permeabilization Solution Kit (BD Biosciences, San Diego, CA) following the manufacturer’s instructions. Afterward, the fixed cells were concentrated twice by centrifugation at 800 rpm for 5 min and subsequent washing with the Buffer BD Perm Wash™. The cells were then stained with the primary antibodies previously described and the proper secondary antibodies. Flow cytometric analyses were carried out in the BD BioSciences, BD FACSVerse, acquiring 10,000 cells. The flow cytometry data were analyzed using the FlowJo version 7.6.2 software (Ashland, OR, USA).

### Microbial Biofilm Formation Single and Mixed on Primary Human Limbo-Corneal Fibroblast Cells

Single (AF, SA) and mixed (AF+SA) biofilm formations were developed using the protocol described by (Ramírez-Granillo et al., 2015), but RPMI 1640-FBS 10% v/v was used as described below.

Three different infection models (AF+HLFC, SA+HLFC, and AF+SA+HLFC) were assayed on HLFC monolayers grown to confluence over 6, 12, and 96 well plates. For the infection process, the cell media were discarded, and the cell monolayers were washed twice using PBS, followed by infection at an MOI of 1 with the microbial inocula previously in culture medium supplemented with FBS as previously described (Luna-Baca et al., 2007; Castañeda-Sanchez et al., 2013). The adhesion phase was left to proceed by incubating the inoculated cultures at 37°C under a 5% CO_2_ atmosphere for 4 h. After the adhesion phase, the culture medium on each well was changed for fresh RPMI 1640+FBS 10% v/v, and the incubation continued until reaching 24 h to achieve the maturation phase of the biofilm (Ramírez Granillo et al., 2015; González-Ramírez et al., 2016). For all assays, a monolayer of uninfected HLFC was used as a control to verify that no significant cell culture changes were detected over time.

### Biofilm Quantification by the Christensen Crystal Violet Method

Single and mixed biofilm cultures with and without HLFCs on 96-well plates (Nunc™, Roskilde, Denmark) were left to proceed for 6, 12, and 24 h. Afterward, the supernatant was discarded, and the biomass produced was evaluated as previously described by Christensen et al. (1985), with the modifications proposed by Ramírez-Granillo et al. (2015). Subsequently, adhered cells were fixed with 99% methanol (200 µl) for 15 min. After removing the methanol, 200 µl of 0.005% crystal violet were added and stained for 20 min. The dye excess was removed and allowed to dry at room temperature. After drying, the contents of the well were washed gently with distilled water. The washes were carried out until the total elimination of the crystal violet reagent. Additionally, to extract the dye absorbed in the biofilm, 200 µl of 33% acetic acid were added, avoiding touching the bottom and walls of the wells. The acetic acid was allowed to act for 15 min. Then, the excess acetic acid was removed and quantified at a wavelength of 595 nm using the ELISA microplate reader Multiskan Ascent Thermo Labsystems. Three individual experiments for each infection model were evaluated.

### Biofilm Metabolic Activity by Tetrazolium Salts Reduction Method

The biofilm metabolic activity was evaluated by the reduction of 3-(4, 5-dimethyl-2-thiazolyl)-2,5-diphenyl-2H-tetrazolium bromide (MTT) as previously described by (Walenka et al. 2005). After the biofilm maturation process (6, 12, and 24 h), the supernatant of the infected cells was discarded, followed by one washing step with PBS. After the washing step, 100 µl of PBS and 100 µl of the MTT solution (SIGMA^®^, St. Louis, MO, USA) at 0.3% were added to each well. The cells were incubated for 2 h at 37°C. After the incubation period, the supernatant on each well was discarded, and 150 µl of dimethyl sulfoxide (DMSO) (Riedel-de Haën™, Seelze, Germany) in 25 µl of glycine buffer (0.1 M, pH 10.2) were added to each well followed by an incubation of 15 min at room temperature under light shaking. Finally, the microplates were read at 540 nm using the ELISA microplate reader Multiskan. Three individual experiments for each infection model (monospecies biofilm and mixed biofilm with HLFCs and without HLFCs) were evaluated.

### Assessment of Biofilm Formation by Scanning Electron Microscopy and Transmission Electron Microscopy

For SEM, HLFC cultures grown on 12-well plates (Santa Cruz Biotechnology, Santa Cruz, CA, USA) were infected as previously described above (monospecies biofilm and mixed biofilm with HLFCs and without HLFCs). After a biofilm maturation period of 24 h, cells were fixed for 2 h with 2% glutaraldehyde (Electron Microscopy Sciences^®^, Washington, PA, USA). Then, cells were washed twice with PBS, and a postfixation step with 1% osmium-tetroxide (Electron Microscopy Sciences^®^, Washington, PA, USA) was carried out, incubating the cells for 2 h. The bases of the wells were removed using a warm metal auger. The samples were placed in a polystyrene plate and dehydrated with subsequent solutions of ethanol (10%–90%) before a final dehydration step with ethyl alcohol 100% for 10 min in triplicate (JT Baker, Phillipsburg, NJ, USA) (Bozzola and Russell, 1999; Vázquez-Nin and Echeverría, 2000). To desiccate samples until the critical point, one drop of hexamethyldisilazane (Electron Microscopy Sciences^®^, Washington, PA, USA) was added to each sample and left to evaporate completely (Hazrin-Chong and Manefield, 2012). Biofilm samples were covered with a gold-palladium ally for 70 s at 5.0 kv. Finally, samples were observed under a high-resolution scanning electron microscope (JEOL, Field Emission Scanning Electron Microscope JSM-7800F, Japan). For TEM, samples were prepared the same way as that for SEM until the ethanol dehydration step, after which the samples were included in resin ON at 60°C for the polymerization step. Semi-fine cuts of the included samples were made with Leica Ultracut UCT (Wetzlar, Germany) equipment and exposed to lead-uranyl solutions for contrast. Finally, samples were mounted for their observation by TEM (JEOL Tokyo, Japan) at Central Microscopy Laboratory of the ENCB-IPN.

### Detection of Biofilm Components by Epifluorescence Microscopy

For the detection of biofilm component by epifluorescence microscopy (EFM), HLFCs were grown over sterile glass coverslips (Velab, Mexico City, Mexico) on 12-well plates. Cells were infected as previously described; fungal biofilm, bacterial biofilm, and mixed biofilm were included. After a biofilm maturation period of 24 h, biofilms were stained with calcofluor white (CW) (1 g/L) (Sigma-Aldrich, St. Louis, MO, USA) for the detection of chitin and N-acetylglucosamine, with Concanavalin A-Alexafluor 488 (ConA) (1 mg/ml) (Sigma-Aldrich St. Louis, MO, USA) for the detection of glucose and mannose residues, while propidium iodide (PI) (10 mg/ml) (AbD Serotec, Raleigh, NC, USA) was used to stain nucleic acids and extracellular DNA. The stained cells were observed under an epifluorescence microscope (Imager.Z2, Apotome 2.0, Carl Zeiss, Germany) at the following wavelengths: CW (λ_Excitation_ = 355 nm; λ_Emission_ = 433 nm); ConA (λ_Excitation_ = 495 nm; λ_Emission_ = 519 nm); PI (λ_Excitation_ = 535 nm; λ_Emission_ = 617 nm). Images were analyzed with the LSM Image Brower version 4.0 software (Carl Zeiss, Germany).

### Statistical Analysis

The data obtained from the three different experiments of biofilm quantification methods were analyzed by two-way ANOVA. To determine the statistical significance of the observed differences, a Student–Newman–Keuls (SNK) was used. For statistical significance, a p-value of <0.05 was used. The values of the means of the different samples in the assays performed were corrected by subtracting the value of the negative control. The negative control used in all experiments was RPMI 1640+FBS 10% v/v. Data were plotted using SigmaPlot 12.0 (Systat Software Inc., San Jose, CA, USA). These characteristics of the statistical analyses were handled according to the recommendations of Allkja et al. (2020).

## Results

### Characterization of the Human Limbo-Corneal Fibroblast Cell Primary Cultures

The phenotypic profile of the HLFC primary cultures was assessed by flow cytometry. Three different markers of limbo-corneal fibroblasts were selected: vimentin (VIM), cytokeratin (CTK), and alfa smooth muscle actin (SMA). The flow cytometric analysis revealed that 99.1% of the cells expressed VIM, while only 7.17% expressed CTK and 8.75% expresses SMA. Negative controls for each marker were also included in the analysis, allowing the corroboration of the HLFC phenotype as VIM^+^CTK^-^SMA^-^ (**Supplementary Figure S1**).

### Biofilm Formation on Human Limbo-Corneal Fibroblast Cells by Christensen Crystal Violet Method

**Figure 1:**
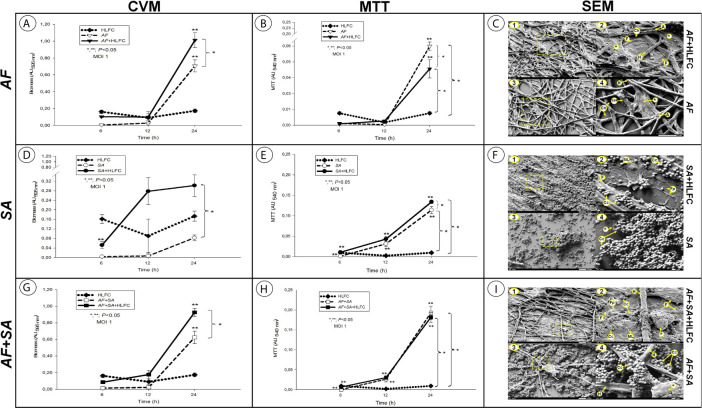
Quantification of microbial biofilms on primary HLFCs. Biomass quantification using the Christensen crystal violet method (CVM). AF **(A)**, SA **(D)**, and the FBI AF+SA **(G)** with and without HLFCs. Metabolic activity quantification using the tetrazolium salt reduction method (MTT) on the biofilm AF **(B)**, SA **(E)**, and AF+SA **(H)** with and without HLFCs. The results are four replicates of three different experiments: n = 12. Significance was determined using the Student–Newman–Keuls test with multicomparison of procedures and are indicated as: (*), p < 0.050. SEM micrographs of biofilms of AF+HLFC (**C1**: 1,000×, **C2**: 2,500×) and AF (**C3**: 1,000×, **C4**: 2,500×); biofilm of SA+HLFC (**F1**: 1,000×, **F2**: 5,000×) and SA (**F3**: 1,000×, **F4**: 5,000×); and biofilm of AF+SA+HLFC (**I1**: 1,000×, **I2**: 5,000×) and AF+SA (**I3**: 1,000×, **I4**: 5,000×). HLFCs, human limbo-corneal fibroblast cells; AF, *Aspergillus fumigatus*; SA, *Staphylococcus aureus*; H, hypha; F, fibroblast; A, anastomosis; Ch, channels; Asterisk (*), extracellular matrix; Eps, exopolymeric substance; ML, monolayer; Fp, filopodia; Cc, cocci.

The amount of biomass was assessed by the CVM under each of the experimental conditions described after 6, 12, and 24 h of biofilm formation. All of the experimental models showed an optimal biomass production at 24 h postinfection. In the AF+HLFC model, the amount of biomass produced at 24 h was higher [absorbance unit (AU) >1.0] in comparison to the biomass produced by the fungal biofilm alone (AU <0.7) (**Figure 1A**). In the SA+HLFC model, an increase in the biomass production was detected (AU >0.3) with respect to the biomass produced in the bacterial biofilm without HLFCs (AU <0.1) (**Figure 1D**). Finally, for the AF+SA+HLFC model, an increase in the biomass production of AU <1.0 was detected in comparison to the mixed biofilm excluding HLFCs in which a statistically significant decrease in the biomass production was observed (AU >0.6) (**Figure 1G**). The uninfected HLFC monolayer used as a control was evaluated, and no significant changes in monolayer biomass over time were detected.

### Metabolic Activity of the Biofilms Formed on Human Limbo-Corneal Fibroblast Cells by MTT

The *in vitro* reduction of tetrazolium salts (MTT) method revealed that the metabolic activity of the sessile cells embedded in the biofilm is optimal. For all the biofilm models, the metabolic activity was determined at 24 h. For the AF+HLFC model, the metabolic activity of the fungal biofilm was AU >0.04, while, compared to monospecies biofilm, the metabolic activity without the HLFC monolayer increased (AU >0.06), which represented a statistically significant difference (**Figure 1B**). We detail this aspect further on, since it describes a possible inhibition mechanism by the HLFCs over *A. fumigatus*. For the SA+HLFC model, an efficient metabolic activity was detected (AU <0.14), which was directly proportional to the incubation time. When comparing the above *S. aureus* biofilm on fibroblasts, bacterial viability was significantly reduced with the bacterial biofilm without HLFCs (AU <0.12) (**Figure 1E**). The metabolic activity for both mixed biofilm models (AF+SA and AF+SA+HLFC) was estimated as the maximum value of AUs (AU >0.20). Compared to the monospecies biofilm models with HLFC and without HLFC, no statistically significant differences were observed between both mixed biofilms in the MTT assay (**Figure 1H**). The uninfected control (HLFCs alone) maintained a basal absorbance.

### Morphological Analysis of Biofilm Formation on Human Limbo-Corneal Fibroblast Cells by Scanning Electron Microscopy and Transmission Electron Microscopy

The biofilms were developed on different surfaces (polystyrene/abiotic and HLFC/biotic). The typical characteristics (specific for each microbial biofilm model) are shown in **Table 1**.

**Table 1:** Results of the morphological analysis of biofilm formation over HLFCs by SEM and TEM.

Biofilms models	Features of biofilm						Figure
	ECM	Hyphae	Anastomosis	Aerial channels	Bacteria	Bacteria microcolonies	
AF+HLFC	++++	++++	++++	✓	X	X	**Fig. 1-C1**
							**Fig. 1-C2**
SA+HLFC	+	X	X	X	+++	+++	**Fig. 1-F1**
							**Fig. 1-F2**
AF+SA+HLFC	+++	+	++	✓	+++	+++	**Fig. 1-I1**
							**Fig. 1-I2**
AF	++	+++	+++	✓	X	X	**Fig. 1-C3**
							**Fig. 1-C4**
SA	+	X	X	X	++++	++++	**Fig. 1-F3**
							**Fig. 1-F4**
AF+SA	+	+	++	✓	+++	+++	**Fig. 1-I3**
							**Fig. 1-I4**

AF, Aspergillus fumigatus; SA, Staphylococcus aureus; HLFC, human limbo-corneal fibroblast cells; ECM, extracellular matrix.

(+), Proportion observed characteristic; (✓), These structures were observed; (x), These structures were not observed.

**Figure 2:**
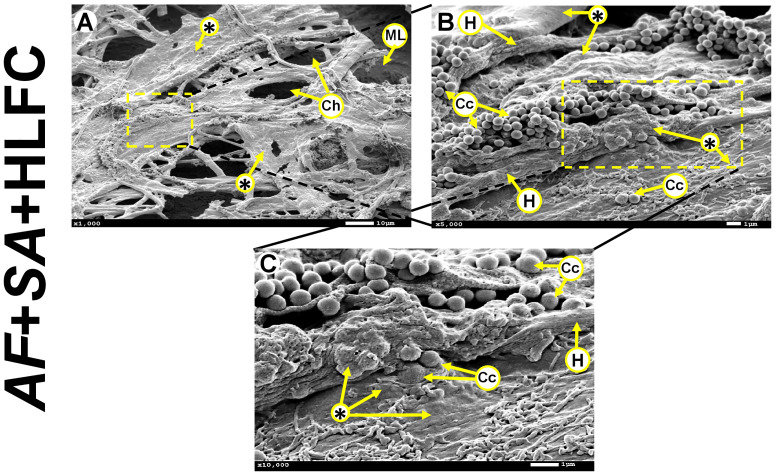
MFBB on primary HLFCs. SEM micrographs of 24-h biofilm of AF+SA at different zooming (**A**: 1,000×; **B**: 5,000×; **C**: 10,000×). ECM air channels and fibroblast monolayer are observed **(A)**. Cocci and hyphae are seen embedded in a condensed extracellular matrix **(B, C)**. MFBB, mixed fungal–bacterial biofilm; HLFCs, human limbo-corneal fibroblast cells; AF, *Aspergillus fumigatus*; SA, *Staphylococcus aureus*; H, hypha; Ch, channels; Asterisk (*), extracellular matrix (ECM); ML, monolayer; Fp, filopodia; Cc, Cocci.

Moreover, the formation of MFBB was observed in the AF+SA+HLFC model (**Figure 2A**) with formation of channels, hyphae development, and bacteria embedded in ECM (**Figures 2B, C**).

**Figure 3:**
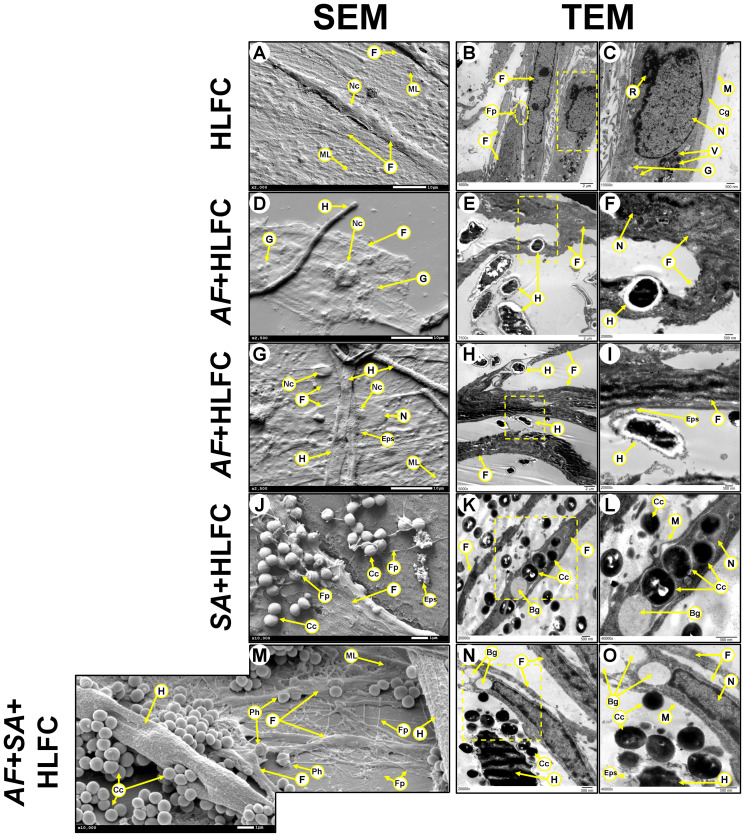
Biofilm formation on HLFCs at 24 h. HLFC: A monolayer by SEM [**(A)**: 2,000×] and its ultrastructure by TEM (**B**: 6,000×; **(C)**: 15,000×x). AF+HLFC: Fungal penetration.(**D**: 2,500×). Hyphae through the fibroblast (**E**: 7,500×; **F**: 20,000×). Fungal adhesion: Secretion of ECM over the HLFC (**G**: 2,500×; **H**: 2,500×; **(I)**: 20,000×). SA+HLFC: The HLFCs form filopodia next to cocci (**J**: 10,000×). TEM showed cocci divided inside the cells. Note the formation of bacterial interstitial (**K**: 20,000×; **(L)**: 40,000×). AF+SA+HLFC: SEM showed cocci affect the hyphae. HLFC forming filopodia. HLFC crescent form and filopodia next to cocci. Fungus appears destroyed (**M**: 10,000×). TEM showed the antibiosis effect over AF. Intracellular damage in the HLFC with the presence of vacuoles (**N**: 20,000×; **O**: 40,000×). HLFCs, human limbo-corneal fibroblast cells; AF, *Aspergillus fumigatus*; SA, *Staphylococcus aureus*; H, hypha; F, fibroblast; ML, monolayer; Fp, filopodia; asterisk (*), extracellular matrix; R, ribosomes; M, cytoplasmic membrane; Cg, collagen fibers; N, nucleus; Nc, nucleolus; V, transfer vesicles; G, secretion granules; Eps, exopolymeric substance; Cc, cocci; Bg, interstitial; Ph, phagocytosis.

The topography of the limbo-corneal fibroblast monolayer without infection under SEM was characterized by a thick flat layer adhered to the abiotic surface. Most of the cells are embedded in an amorphous material (**Figure 3A**). Fibroblasts showed a large fusiform morphology with a length of 170 µm, a concave zone related to the nuclear zone, as well as a convex zone resembling nucleoli. In some borders of the cellular membrane, filopodia could be observed (**Figure 3A**). When the monolayer of HLFCs was observed by TEM, residues of an amorphous material were observable. Nascent filopodia were also detected in the cellular membrane of some fibroblasts (**Figure 3B**). Elongated cells were distributed in palisades with secreted material surrounding their cytoplasmic membrane. The intracellular structures were unmodified. The nucleus showed a size ≈10 µm, with a highly electrodense and well-delimited elliptical nuclear membrane. Also, a circular nucleolus was observed inside the nucleus, with a diameter around 1,500 nm. The cytoplasm was unaltered, and several cellular structures were observed, such as ribosomes (≈2,500 nm), intracellular vesicles (500 nm), and secretory granules (<500 nm) (**Figure 3C**). The cytoplasmic membrane in the HLFCs was unaltered, and collagen fibers adjacent to the outer nuclear envelope were observed.

At 24 h of AF+HLFC biofilm formation, both types of electron microscopy revealed that hyphae are capable of generating cellular damage by two mechanisms. The first fungal process is the penetration of hyphae through the HLFCs by SEM (**Figure 3D**) and TEM (**Figures 3E, F**). The second fungal process is the colonization to the cell surface accompanied by secretion of some extracellular polymeric substances (EPS) (**Figures 3G–I**). This phenomenon did not cause the loss of the nuclear envelope of the fibroblasts.

In the SA+HLFC model, several indicators of cell damage were revealed by SEM, as well as cracks on the surface of the limbo-corneal fibroblast cells that maintained their original size. Cocci were detected on the HLFC monolayer next to the filopodia (**Figure 3J**). By TEM, the bacterial population at the periphery of the HLFCs was observed. Inside the cytoplasm, highly electrodense circular structures were observable (**Figure 3K**). At a higher magnification, as single bacterium was identified. In some cocci, the bacterial septum was evident, indicating cell duplication (**Figure 3L**). Moreover, several interstitial (<1.0 µm in diameter) were detected, and destabilization of the cell membrane caused abnormalities in the cytoplasm, while the nucleolus showed an irregular shape (**Figure 3L**) compared to uninfected cells.

During SEM observation of the mixed biofilm (AF+SA+HLFC), it was shown that the fungal population decreased. In addition, the increase of amorphous fungal structures and the absence of conidia were evident. The affinity of bacteria for *A. fumigatus* was evident compared to HLFCs. Also, apparent damage caused by the bacteria to the fungal wall by coating the hyphae was distinguished. As for fibroblasts, only alterations in shape and size were observed. In fields where the fungus was not perceived, the bacteria were closer to the fibroblasts that produce several filopodia (**Figure 3M**). In the micrographs obtained by TEM, the effect of FBI on HLFCs was similar (**Figure 3N**). The cellular wall of the hyphae secreted EPS that are surrounded by cocci. In the same field, an HLFC showing an interstitial, but still with an unaltered nucleus, could be observed (**Figure 3O**). When we observed other fields, it was possible to observe that *S. aureus* was able to infect HLFCs and cause cell lysis. However, in certain fields, the fibroblasts produce filopodia appearing to surround cocci (**Figure 3J**).

**Figure 4:**
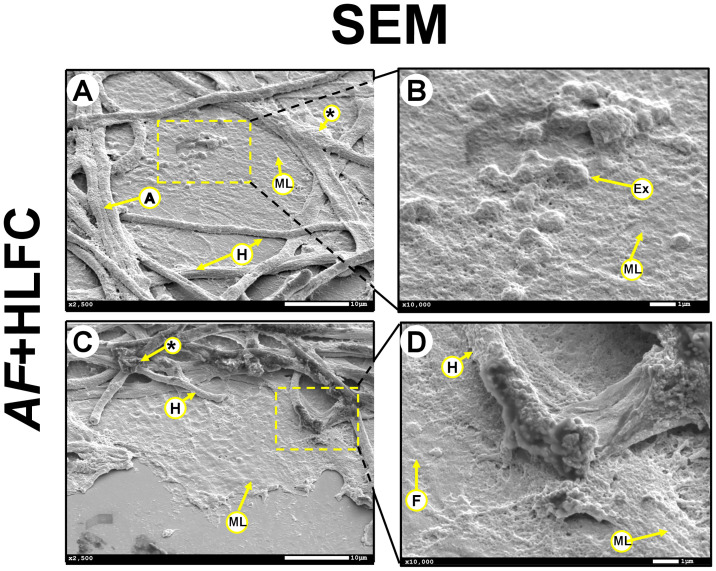
Interaction between AF biofilm and HLFCs. SEM micrographs showed an antifungal effect caused by the HLFC at 24 h. The HLFC showed exosome-like structures that produce damage to the hyphae (**A**: 2,500×; **B**: 10,000×). Hypha damaged in the apical zone. (**C**: 2,500×; **D**: 10,000×). HLFCs, human limbo-corneal fibroblast cells; AF, *Aspergillus fumigatus*; H, hypha; F, fibroblast; A, anastomosis; asterisk (*), extracellular matrix; Ex, exosome like; ML, monolayer.

Throughout the course of the fungal biofilm on HLFCs, severe damage to the hyphae was observed. In some fields, the hyphae retracted while colonizing the cellular surface (**Figure 4A**). During this interaction, spherical bodies, with a diameter of ≈100 nm, were detected over the cellular monolayer; these structures could be secretory granules or exosomes (**Figure 4B**). In other fields, the fungi were able to colonize and degrade the cell monolayer; hyphae were observed enveloped in a dense material (**Figure 4C**). The hyphae showed a scalded appearance and were abruptly terminated in the apical zone (**Figure 4D**).

### Biofilm Fungal Bacterial Composition on Primary Human Limbo-Corneal Fibroblast Cells Using Epifluorescence Microscopy

**Figure 5:**
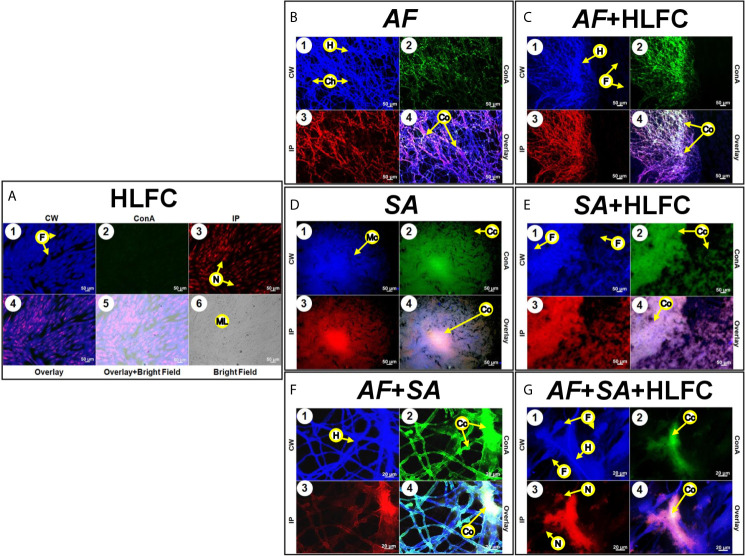
Molecular biofilm components detected on HLFCs. EFM micrograph at 24 h showed ECM composition over the HLFC monolayer. HLFC (**A**: 10×), AF (**B**: 10×), AF+HLFC (**C**: 10×), hyphal network and co-localization of carbohydrates and eDNA in the ECM. SA (**D**: 10×), SA+HLFC (**E**: 10×), co-localization of carbohydrates and eDNA in the center of the bacterial microcolonies growing over the fibroblasts. AF+SA (**F**: 40×) and AF+SA+HLFC (**G**: 40×) showed antibiosis against AF for both. Calcofluor White (CW: blue-chitin and glycosylated carbohydrates), concanavalin A (ConA: green-glucose and mannose residues), and propidium iodide (PI: red-nucleic acids). Co-localization was obtained by image merging. HLFCs, human limbo-corneal fibroblast cells; AF, *Aspergillus fumigatus*; SA, *Staphylococcus aureus*; H, hypha; F, fibroblast; Ch, channels; ML, monolayer; Cc, cocci; Co, co-localization; Mc, microcolony; N, nucleus.

**Figure 6:**
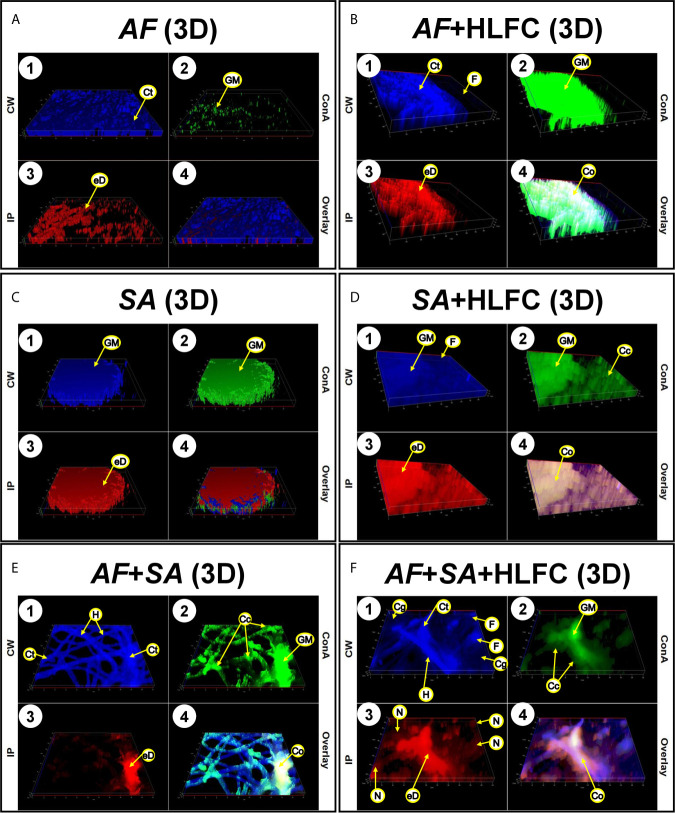
Construction of 3D models of the biofilms on HLFCs. AF **(A)**: a biofilm rich in carbohydrates was detected. AF+HLFC **(B)**: biofilm is denser with strong co-localization of the ECM components on the monolayer. In the bacterial 3D model, microcolonies are enveloped in layers of polysaccharides and eDNA, with stronger co-localization in SA+HLFC **(D)** compared with SA **(C)**. The ECM was scarce for FBI models, AF+SA **(E)**, and AF+SA+HLFC **(F)**, detection of a strong antibiosis over AF. Calcofluor White (CW: blue-chitin and glycosylated carbohydrates), concanavalin A (ConA: green-glucose and mannose residues), and propidium iodide (PI: red-nucleic acids). Co-localization was obtained by image merging. HLFCs, human limbo-corneal fibroblast cells; AF, *Aspergillus fumigatus*; SA, *Staphylococcus aureus*; Ct, chitin; GM, N-acetylglucosamine/glucose and mannose residues; eD, extracellular DNA; F, fibroblast; Co, co-localization; H, hypha; Cc, cocci; N, nucleus.

Uninfected HLFC monolayers were analyzed by EFM using several dyes to detect ECM components as well as chitin-like compounds in the monolayer (**Figure 5A1**). Furthermore, the detection of carbohydrate residues was weak (**Figure 5A2**), and PI clearly showed the nuclei of the fibroblast, but not extracellular DNA (eDNA) (**Figure 5A3**). Bright-field images of the cell monolayer (**Figure 5A6**) showed a flat surface covered by a dense material identified as carbohydrate (**Figure 5A5**). Respectively, in fungal biofilms with HLFC and without HLFC, higher ECM production by *A. fumigatus* on fibroblasts was confirmed. The highest signal was observed in the AF+HLFC biofilm, with all three dyes (**Figure 5C**) demonstrating that the ECM composition is chitin, glucose, and/or mannose residues and eDNA (**Figures 5B, C**). Likewise, fibroblasts were weakly labeled with CW (**Figure 5C1**). However, hyphae were strongly marked; a similar effect occurred with ConA (**Figure 5C2**). In the 3D model of fungal biofilm with HLFC, increased fluorescence was observed in the biofilm structures (**Figure 6B**) compared to the monospecific biofilm (**Figure 6A**). In addition, a higher amount of glucose or mannose (**Figure 6B2**) and eDNA (**Figure 6B3**) was detected in AF+HLFC than in the fungal biofilm without fibroblasts (**Figures 6B3, A2, A3**). Moreover, a co-localization effect was observed with dense fluorescence and structural integrity of the fungal biofilm developed on the HLFC monolayer (**Figure 6B4**). On the other hand, the composition of the ECM was similar in the bacterial biofilms, with carbohydrates and eDNA being the main components (**Figures 5E, D**). In the 3D structure of the biofilm, it was observed that the radius of the microcolonies is larger during colonization of the cell monolayer (**Figure 6D**). This HLFC monolayer is still organized (**Figure 6D1**) but with abundant cocci surrounding the cells. Also, eDNA and carbohydrates are in the center of the bacterial microcolonies in both models (**Figures 6C4, D4**). During FBIs, CW showed the highest labeling for the hyphae (**Figures 5F, G**) despite the reduction in these fungal structures in mixed biofilm including HLFC (**Figure 5G**) and a high number of bacteria surrounding the hyphae; these cocci were mainly marked by ConA (**Figure 5G2**); eDNA detection is evident on the hyphae (**Figure 5G3**). Furthermore, 3D constructions corroborated that hyphae are surrounded by numerous cocci and are scarce in the AF+SA+HLFC model (**Figures 6E, F**). Additionally, in the merged images, the co-localization of eDNA and complex and simple carbohydrates is evident (**Figures 6E4, F4**).

### Interaction Model During Mixed Infection on *In Vitro* Human Limbo-Corneal Fibroblast Cell Culture

**Figure 7:**
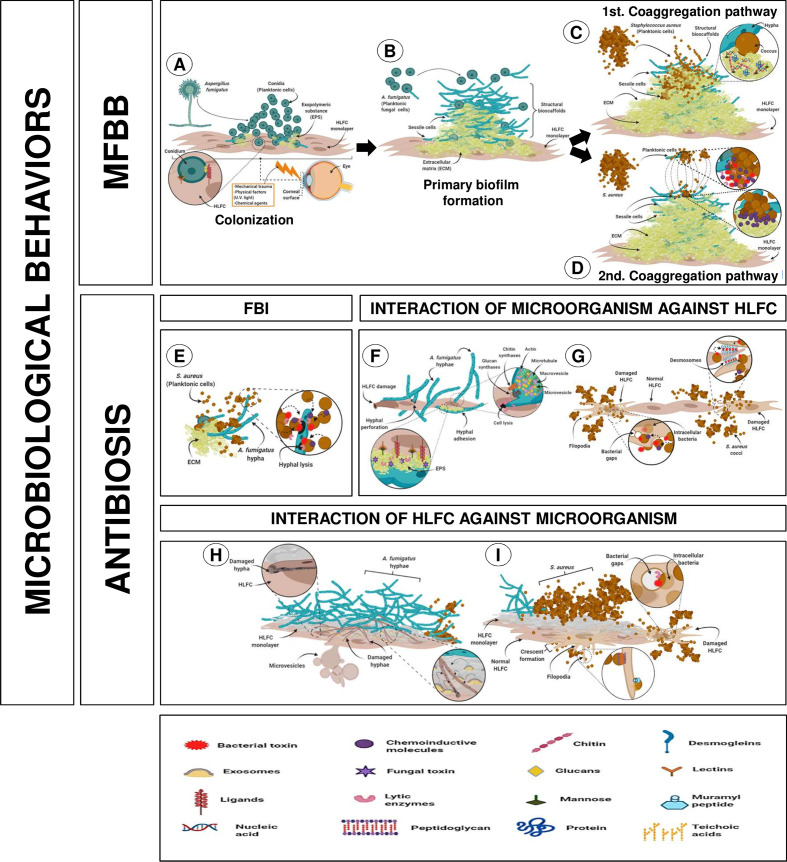
Putative interaction of mixed infection on HLFCs related to eye infections. [MFBB] **(A)** Colonization of surface HLFCs by planktonic conidia. **(B)** A primary biofilm with rigid and abundant ECM, with planktonic propagules. Co-aggregation pathway could follow two different routes. **(C)** First pathway: secondary colonizer forms new bioscaffolds of sessile cocci. **(D)** Second pathway: planktonic bacteria induce co-aggregation over the fungal biofilm, reaching the sessile stage. Non-aggregate planktonic cells can migrate to another site. [ANTIBIOSIS] **(E)** Antagonistic effect from *S. aureus* over *A. fumigatus*. MICROORGANISMS AGAINST HLFC. **(F)** *A. fumigatus* causes hyphal perforation and EPS secretion, and hyphal adhesion. **(G)** *S. aureus* triggers pores and cellular lysis affecting membranes and cytoskeleton, breaking the desmosomes. HLFC AGAINST MICROORGANISMS. **(H)** HLFC against AF: Fibroblast produces exosomes and microvesicles that could be involved in phagocytic processes. **(I)** HLFC against SA: Fibroblast projects filopodia surrounding the cocci that are accompanied by modifications of the cytoskeleton (crescent formation). For details, see the *Results* section. Created with BioRender.com.

The set of results obtained in this study provided a backdrop to describe the possible events that occur during the establishment of mixed biofilms on HLFCs. Therefore, a graphical overview was constructed for understanding these microbial effects through three different pathways (**Figure 7**). The data suggest that possibly two microbiological behaviors were detected during the FBI.

The first behavior was MFBBs, which initiates with the colonization of the monolayer surface by planktonic conidia, maintaining the stable union of the fungal surface with the HLFCs (**Figure 7A**). At this stage, secretion of EPS promotes the ECM formation. A mature fungal biofilm was characterized by an abundant and rigid ECM; planktonic propagules contribute to form structural bioscaffolds that reach a sessile stage. The co-aggregation pathway could follow two different routes (**Figure 7B**). In the first pathway, the secondary colonizer joins the surface of the mature fungal biofilm, forming new bioscaffolds of sessile cocci (**Figure 7C**). In the second pathway, planktonic bacteria induce co-aggregation in the fungal biofilm, reaching the sessile phase. At this point, it is possible that these unaggregated planktonic cells can then migrate to another site (**Figure 7D**).

On the other hand, the second behavior was antibiosis relationships. During FBI, *S. aureus* inhibits *A. fumigatus*, which may be involved in the production of unknown compounds that trigger cell lysis (**Figure 7E**).

Regarding *A. fumigatus* against HLFCs, there are two possible ways for fungus spreading. The first is by hyphal perforation [turgor mechanisms accompanied by the performance of the Spitzenkörper system (Cell wall enzymes, microvesicles, and macrovesicles) as well as thigmotropic reactions] and the second by EPS secretion and hyphal adhesion (**Figure 7F**). Furthermore, the behavior of *S. aureus* against limbo-corneal fibroblasts led to the dissemination of the bacterium into the human cell, triggering pore formation and cell lysis; this behavior affects cytoplasmic membrane and the cytoskeleton with disruption in desmosomes (**Figure 7G**). Finally, the effect of HLFCs against microbes is described, where a self-defense conducted through various innate immune mechanisms is triggered (exosomes, phagocytic microvesicles, and crescent formation) (**Figures 7H, I**).

## Discussion

The processes and factors involved in the establishment of polymicrobial biofilms remain poorly characterized. In the case of MFBBs, several studies suggest that FBIs are driven by physical interactions between the biofilm components. An important example of this type of relationship occurs between two of the main etiologic agents of microbial keratitis worldwide: *A. fumigatus* and *S. aureus*.

Previous studies have suggested that the interaction between these two microorganisms can form biofilms over abiotic surfaces (Ramírez Granillo et al., 2015); in fact, the methodologies used were quite similar, but in this study, we used RPMI supplemented with FBS to have the same conditions when culturing fibroblasts. The results of this research demonstrated that this fungus and bacteria are capable of forming biofilms on biotic surfaces. Additionally, to our knowledge, this is the first report where *A. fumigatus*–*S. aureus* interaction has been observed in a primary cell culture of HLFCs with biofilm formation.

In general, the results showed that biofilm development is more efficient on biotic surfaces (HLFCs) than on abiotic surfaces (polystyrene); to directly compare both surfaces, FBS was added to all treatments (**Figure 1**, **Table 1**). Similarly, abundant amounts of extracellular material of single and mixed biofilms were demonstrated in the presence of the HLFC monolayers by EFM (**Figures 5**, **6**). This qualitative technique of biofilm has been used by our research group for the detection of biomolecules that constitute the ECM (Ramírez Granillo et al., 2015; González-Ramírez et al., 2016; Camarillo-Márquez et al., 2018; Bautista-Hernández et al., 2019). Other authors have reported that by using molecules to specifically eliminate ECM components, such as sodium periodate (that destroys carbohydrates), DNase (that digests DNA), and proteinase K (that digests proteins), it was possible to demonstrate that fluorochromes detect specifically such biomolecules (Baillie and Douglas, 2000; Chandra et al., 2001; Córdova-Alcántara et al., 2019). This biofilm detachment assay represents a good approximation to the composition of ECM. However, some authors have noted that not all the components are available or are susceptible to the action of the degrading molecules; for example, the oxidation of carbohydrates is not fully accomplished as demonstrated by Ikeda et al. (2007) and Doern et al. (2009).

When the human ocular surface constituted by HLFCs is compromised by mechanical damage, adhesion sites are exposed, generating an optimal environment for adhesion and establishment of microbial populations in the eye. Likewise, on abiotic surfaces, the adhesion processes are nonspecific and are mediated by hydrophobic and electrostatic forces. This is demonstrated by the reversibility of the adhesion process on abiotic surfaces not pretreated with synthetic substrates, microorganisms, or tissues known to favor microbial adhesion (Rittman, 1989; Asaria et al., 1999; Fulcher et al., 2001; Dunne, et al., 2002; Harris et al., 2002; Parsa et al., 2010; Sun et al., 2010; Percival et al., 2011; Abelson and McLaughlin, 2012; Sengupta et al., 2012; Zhang et al., 2012; Samimi et al., 2013; Bispo et al., 2015; Boukahil and Czuprynski, 2018; Ponce-Angulo et al., 2020). Thus, the development of mixed biofilms on abiotic surfaces previously conditioned with primary cell cultures is an opportunity to understand the features and processes of such polymicrobial associations. The intention to obtain a close eye-like response was the main reason why we chose primary cultures of HLFCs in this work. Moreover, it is well known that primary cultures had a finite number of duplications and had characteristics closer to the original host. We do not use cell lines because of their aneuploidy; cell lines had lost the original host characteristics.

On the other hand, we used the biomass quantification and metabolic activity determination to understand the ecological relationships between our three microbial models. When the HLFC culture was analyzed, neither the biomass production nor the metabolic activity of the culture was modified during the kinetics performed (**Supplementary Figure S2A**). These basal lectures indicated that both CVM and MTT techniques are sufficiently sensitive to detect the microorganisms in the biofilm experiments (Mowat et al., 2007; Ramage et al., 2009; Camarillo-Márquez et al., 2018). Besides, the mixed biofilm and fungal biofilm with HLFCs produced the highest amount of biomass, while the bacterial biofilm on limbo-corneal fibroblasts produced significantly less. These results are similar to those reported for our research group on an *in vitro* mixed biofilm, with the exception of MTT assays (Ramírez Granillo et al., 2015). We reported that *A. fumigatus* establishes a dense biofilm. This fungus is a better biofilm producer than *S. aureus* over abiotic surface; the same was confirmed on biotic surfaces. Therefore, we suggest that the formation of MFBB on HLFCs begins with the interaction of the conidium and limbo-corneal fibroblasts, leading to the expression of several molecular components that allow an initial stable adhesion, giving rise to the beginning of the colonization process of the biotic surface. These planktonic fungal populations (conidium and hyphae) adhere consecutively origination “structural bioscaffolds”, which are exploited by Gram-positive bacteria. After the cocci adhesion, bacterial aggregates appeared, and a true mixed biofilm is formed on HLFCs. This hypothesis needs to be tested in more detail to characterize the molecules that drive the process, leading to the identification of possible therapeutic targets that could aid in the treatment of polymicrobial keratitis.

In contrast, the MTT reduction assays (**Supplementary Figure S2B**) also allowed the identification of a second microbial behavior: “the antibiosis relationship for this FBI”. The maximum absorbance value was for the mixed biofilm over HLFCs, followed by the bacterial biofilm, and finally the fungal biofilm. Likewise, the antibiosis effect was observable by SEM; bacteria are the predominant population during this microbial interaction on limbo-corneal fibroblast cultures (**Supplementary Figure S2F**), while the *A. fumigatus* and HLFCs appear diminished in number in the micrographs. Likewise, monolayer destruction is evident and the HLFCs express numerous filopodia in the cellular membrane. Additionally, the size of the fibroblasts is altered (≈10–30 µm), and the monolayer of the HLFCs showed a dramatic erosion from the abiotic surface. These results are consistent with a previous report from our research team (Ramírez Granillo et al., 2015) in which the antibiosis on this filamentous fungus by the action of *S. aureus* during biofilm formation *in vitro* was reported. This mycophagy event, generated by the bacteria in the HLFC model, has a direct impact over the fungal population, since bacteria are taking advantage of the fungal components for self-nutrition (Leveau and Preston, 2008). Mycophagy events have previously been reported for this S*taphylococcus* species in other *in vitro* fungal–bacterial models (Ikeda et al., 2007; Camarillo-Márquez et al., 2018; Bautista-Hernández et al., 2019). In summary, the correlation of the MTT values with the microscopic evidence obtained by SEM suggests that in this FBI on HLFCs, the prevalent microorganism is *S. aureus*.

In this work, several antibiosis effects were detected, suggesting a microbial war taking place in at least three fronts: FBI (AF-SA: antagonistic relationship previously described), microbial interactions (AF, SA, AF-SA) with HLFCs, and interactions between HLFCs against microorganisms (**Figure 7**).

The fungal antagonism effect against the limbo-corneal fibroblast cells was also evidenced by SEM and TEM for the assayed models. The first fungal antibiosis effect is related to hyphal perforation of HLFCs (**Figures 3D–F**). Hyphal perforation of pulmonary endothelial cells by *A. fumigatus* has previously been reported and has been associated with the disruption of the endothelial barrier to promote *in vivo* hematogenous dissemination of the fungi (Kamai et al., 2009). Non-mechanical perforation mediated by the turgor of the apical zone of the hyphae has also been described, accompanied by the Spitzenkörper process that causes the accumulation on vacuoles filled with lytic enzymes. Likewise, thigmotropic reactions that deform the cell wall are associated with this mechanism (Bowen et al., 2007; Brand and Gow, 2009; Steinberg et al., 2017; Dimou and Diallinas, 2020). The other type of fungal damage is the secretion of EPS with subsequent adhesion of the hyphae, also detected in this work over the cell culture (**Figures 3G–I**). Previous studies of pulmonary epithelia of patients infected with *A. fumigatus* have suggested that this fungus is capable of secreting metabolites such as sialic acid by conidia, which are beneficial for the invasiveness process. Sialic acid mediates the adhesion of the fungi to cellular components such as fibronectin and laminin. *Aspergillus* species can secrete other metabolites such as gliotoxins, fumagillin, and several types of proteases that can trigger changes in the cellular membrane and the cytoskeleton to facilitate the invasion process (Dagenais and Keller, 2009; Osherov, 2012; Croft et al., 2016; Gago et al., 2018). We recommend that further studies address the effect of these secondary metabolites and ligands on keratitis, since their participation during this infectious process in the eye has not been studied in detail.

Bacterial antibiosis was related to intracellular invasion, observed in the SA+HLFC model. This behavior was observed in more detail with TEM during FBI (**Figures 3K, L**). Several *in vitro* studies using osteoclasts, endothelial cells, and fibroblasts have suggested that *S. aureus* can act as a facultative intracellular bacterium; this phenomenon has not been observed *in vivo*. The most studied mechanisms involved in the intracellular dissemination of this bacterium *in vitro* are the interactions between *S. aureus* and adhesins related to toxin synthesis. In fact, several lines of evidence suggest that fibronectin in the HLFCs plays an important role during *in vitro* infections and can be related to the keratitis caused by this microorganism (Lowy, 2000; Jett and Gilmore, 2002; Foster et al., 2014; Rollin et al., 2017).

Both the formation of bacterial interstitial and hyphal perforation (**Figures 3M–O**) were observed in the FBI+HLFC biofilm, specifically intracellular spread was also detected by EFM. This interstitial with a diameter <1.0 µm similar to the size of bacteria is observed on the surface of the fibroblasts. When overlaying the images (ConA: green halos; PI: red halos), the bacteria were distinguished as intense orange marks adjacent to the HLFCs; by TEM, it seems to be the same bacterial invasion (**Supplementary Figure S3**). Additionally, changes in cell–cell junctions, particularly in structures that resemble desmosomes, were observed. This phenomenon has been related to several *S. aureus* toxins, such as the Exfoliative Toxin A (ETA), which acts directly over desmoglein, one of the main components of desmosomes (Lowy, 2000; Kowalczyk and Green, 2013; Johnson et al., 2014; Mariutti et al., 2017). Some of our observations suggest a damage in the desmosomes (**Supplementary Figures S4C, D**), as reported during *P. aeruginosa* monoinfections over corneal epithelial cell cultures (Fleiszig et al., 1996; Lee et al., 1999).

On the other hand, there is the antibiosis caused by HLFCs against microorganisms. HLFCs have a direct impact on fungal growth, as evidenced by the presence of thin, poorly branched hyphae in several fields (**Figures 4C, D**). Furthermore, the presence of exosome-like structures in the cytoplasm of the infected HLFCs (**Figures 4A, B**) could be associated with pro-inflammatory immune responses, as has been reported for this type of cells during mycobacterial infections (Castañeda-Sanchez et al., 2013). HLFC-producing microvesicles, which have also been reported during murine fibroblast infections and have been associated with phagocytosis, were also observed (Masci et al., 2016). Thus, for fungal dissemination, we could suppose that these structures represent some type of defense mechanism against this filamentous fungus agent (**Supplementary Figures S4E, F**).

Several structures in the fibroblast monolayer were visualized throughout the micrographs observed in the SA+HLFC model. These resemble the cellular structures associated with innate immune responses, such as filopodia surrounding cocci (**Figure 3J**), which in other cellular models have been associated with phagocytic processes of bacterial agents mediated by Toll-like receptors. Similarly, HLFCs showed crescent formation (**Supplementary Figures S4A, B**) during this FBI on monolayers. We suggest that this behavior was related to a phagocytosis process. These phenomena together with the filopodia (**Figure 3M**) and microvesicles (**Supplementary Figure S4C**) have been found to suggest a phagocytic activity by the limbo-corneal fibroblast cells, as reported for macrophages. This behavior could be directly related to the immunology of the eye, which is considered a privileged immune organ with an innate response (Kress et al., 2007; Heimer et al., 2010; Pérez et al., 2013; Jimenez-Martinez et al., 2016; Masci et al., 2016; Bautista-Hernández et al., 2017; Horsthemke et al., 2017; Rosales and Uribe-Querol, 2017). These ideas allowed us to represent the possible events that occur during the MFBB over the HLFCs (**Figure 7**).

In this study, we were able to identify first evidence on the MFBB of potential opportunistic pathogens (*A. fumigatus*–*S. aureus*) and ocular host response (HLFC). In addition, our data suggest that both microbial agents are able to attack and destroy the limbo-corneal fibroblast cell monolayers, but the HLFCs are able to strike back. Furthermore, our results suggest a microbial warfare on three different fronts. The first is a clear antibiosis between *A. fumigatus* and *S. aureus*. The second and third are a bidirectional antibiosis between microorganisms against fibroblasts. We believe that our experimentation could open a new research field to understand the eye immunology and its interactions with biofilm polymicrobial infections. The ecological interactions are complex, and all of the members interact with each other. Further understanding could permit the use of this microbial warfare as a source of new therapeutic molecules.

## Data Availability Statement

The raw data supporting the conclusions of this article will be made available by the authors, without undue reservation.

## Author Contributions

AR-G performed key experiments and drafted the manuscript. LAB-H, FSM-G, AD-L, and IMC-A worked in the laboratory with the cell cultures and biofilm formation and quantification. VMB-D, NOP and MAM-R participated in the experimental design and paper edition and discussions. AVR-T is the leader of the research line and funded the investigation. All authors contributed to the article and approved the submitted version.

## Funding

We thank the Research and Postgraduate Secretariat ENCB-IPN and the COFAA grant of the Instituto Politécnico Nacional, Mexico City, for financial support [SIP20195841, SIP20195630, SIP2020172, and 20210200].

## Conflict of Interest

The authors declare that the research was conducted in the absence of any commercial or financial relationships that could be construed as a potential conflict of interest.

## Publisher’s Note

All claims expressed in this article are solely those of the authors and do not necessarily represent those of their affiliated organizations, or those of the publisher, the editors and the reviewers. Any product that may be evaluated in this article, or claim that may be made by its manufacturer, is not guaranteed or endorsed by the publisher.

## Supplementary Material

Supplementary Figure 1 Immune typification of the primary *in vitro* culture of HLFC. Flow cytometry analysis is representative for a population of 100,000 cells from a population of a 66.2% subset of the cell lineage **(A)**. Anti-rabbit **(B, H)** and Anti-Mouse **(E)** were used as negative controls. It was observed that antibodies directed for Vimentin (VIM) were expressed with more than 99% **(C)**, whereas for Cytokeratin (CTK) **(F)** and alpha smooth muscle actin (SMA) **(I)**, were poorly expressed demonstrating the absence of these markers in the study phenotype. The overlapping of the expression of the markers corroborated the identity of these proteins **(D, G, J)**.

Supplementary Figure 2 Comparison of the analysis of the quantification and characterization methods of monoculture and mixed biofilms and MFBB on HLFC cultures. CVM) AF+HLFC and AF+SA+HLFC showed the highest biomass production (≈1.0 AU); followed by SA+HLFC (≈0.3 AU) which presented a lower biomass production (**A**: TTM) AF+SA+HLFC showed the most efficient metabolic activity (<0.20 AU); followed by SA+HLFC (<0.15 AU) and AF+HLFC (<0.05 AU) (**B**: SEM) The HLFC cultures infection free (**C**: 1000x; 2000x) were observed without apparent alteration (normal size). Observation of the AF+HLFC model showed that hyphae produced a ECM although with a worn-out appearance in some of them. The HLFCs were observed abnormal in size and shape (**D**: 1000x; 2500x). Model SA+HLFC exhibits a few microcolonies formation with EPS production. HLFCs have a normal size, with a presence of surface cracks and developed filopodia (**E**: 1000x; 5000x). The micrographs of the AF+SA+HLFC model reveal that the bacteria is exceeding its growth compared to fungus and fibroblasts. Moreover, the monolayer was limited to certain areas and the HLFCs were observed abnormal on several fields (**F**: 1000x; 5000x). The results are four replicates of three different experiments: n=12. Significance was determined using the Student-Newman-Keuls test, with multicomparison of procedures and are indicated as: (*), P<0.050. HLFCs, Human Limbo-Corneal Fibroblast cells; AF, *Aspergillus fumigatus*; SA, *Staphylococcus aureus*; H, hypha; F, Fibroblast; A, Anastomosis; Ch, Channels; Asterisk (*): Extracellular matrix; ML, Monolayer; Fp, Filopodia; Cc, Cocci.

Supplementary Figure 3 Intracellular infection during FBI on HLFC. Biofilms were grown throughout 24 h on *in vitro* monolayer cultures of HLFCs. EFM) Fibroblasts were detected with CW (A: 63x). Stained Bacteria Con A) enveloping fibroblasts (B: 63x). The IP (C: 63x) showed the nucleus of the HLFCs as red halos. The overlay of the images shows that the bacteria are distinguished as intense orange marks surrounding the HLFCs (D: 63x). A digital zoom showed interstitial of around 1 µm (D1; D2). TEM. AF+SA+HLFC: Fungal population was reduced compared to the cocci surrounding HLFCs (E: 20000x). In the cytoplasm of HLFCs intracellular cocci were observed; fibroblast formed interstitial (<1.0 µm) (F: 40000x). HLFC: Cells without infection were seen with their cytoplasm and internal structures unaltered. In addition, intracytoplasmic inclusions approximate size of 0.3-0.5 µm was observed (G: 6000x; H: 20000x). SA+HLFC: High bacterial population compare to fibroblasts. In the center of the micrograph, a HLFC with interstitial caused by intracellular infection of SA. (I: 15000x). AF+HLFC: Hyphae were seen secreting extracellular material between a group of fibroblasts (J: 7500x). In this model, HLFCs with interstitial were not observed; and some cellular structures can be observed within the cytoplasm (K: 20000x). AF+SA: During this FBI, it is possible to observe abnormal hyphae with polar invaginations, as well as adjacent cocci that attach to the fungal cell wall (L: 20000x). HLFC, Human Limbo-Corneal Fibroblasts; AF, *Aspergillus fumigatus*; SA, *Staphylococcus aureus*; F, Fibroblast; H, Hypha; Cc, Cocci; Co, Co-localization; Bg, Interstitial; Fp, Filopodia; Eps, Exopolymeric substance; Bs, Bacterial septum; M, Cytoplasmic membrane; N, Nucleus; Nc, Nucleolus; Ci, Cytoplasmic inclusions; Mv, Microvesicles.

Supplementary Figure 4 Behavior of HLFC during FBI observed by TEM at 24 h. Intracellular cocci dividing (presence of bacterial septum) a crescent formation is distinguished (**A**: 20000x; **B**: 20000x). Cocci surrounded deformed hyphae (**C**: 15000x). Zooming permitted to observe desmosomes (**D**: 40000x). AF infection caused heterogeneous cytoplasm and adjacent microvesicles to the cytoplasmic membrane of HLFC (**E**: 7500x; **F**: 20000x); hyphae perforate the cytoplasmic membrane. HLFC, Human Limbo-Corneal Fibroblasts; AF, *Aspergillus fumigatus*; SA, *Staphylococcus aureus*; H, hypha; F, Fibroblast; M, Cytoplasmic membrane; Nm, Nuclear membrane; N, Nucleus; Nc, Nucleolus; Eps, Exopolymeric substance; Cc, Cocci; Bg, Interstitial; Bs, Bacterial septum; Mv, Microvesicles; Cf, Crescent formation; D, Desmosomes.

## Abbreviations

ECM, extracellular matrix; FBI, fungal–bacterial interaction; MFBB, mixed fungal–bacterial biofilm; HLFCs, human limbo-corneal fibroblast cells; AF, *Aspergillus fumigatus*; SA, *Staphylococcus aureus*; EPS, extracellular polymeric substances; SEM, scanning electron microscopy; TEM, transmission electron microscopy; EFM, epifluorescence microscopy; CW, Calcofluor White; ConA, concanavalin A; PI, propidium iodide; CVM, Christensen crystal violet method; MTT, tetrazolium salt reduction method; AU, absorbance units; eDNA, extracellular DNA.
