# Supplementary figures and images for: Microbial Warfare on Three Fronts: Mixed Biofilm of Aspergillus fumigatus and Staphylococcus aureus on Primary Cultures of Human Limbo-Corneal Fibroblasts
Source: Front Cell Infect Microbiol. 2021 Aug 16;11:646054. doi: 10.3389/fcimb.2021.646054 (PMC8415486; doi:10.3389/fcimb.2021.646054)

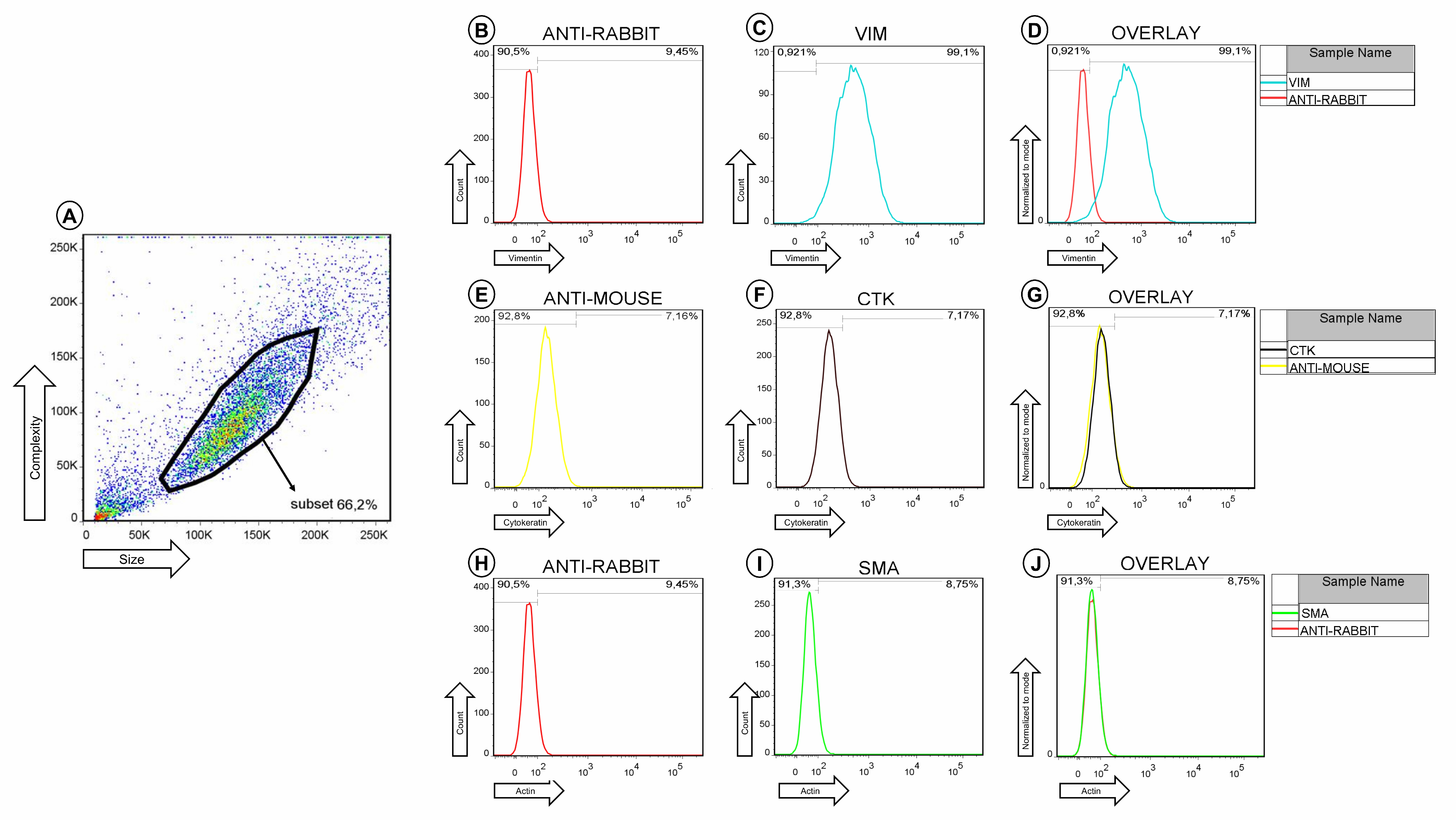

Supplement: Supplementary Figure 1 — Immune typification of the primary in vitro culture of HLFC. Flow cytometry analysis is representative for a population of 100,000 cells from a population of a 66.2% subset of the cell lineage (A). Anti-rabbit (B, H) and Anti-Mouse (E) were used as negative controls. It was observed that antibodies directed for Vimentin (VIM) were expressed with more than 99% (C), whereas for Cytokeratin (CTK) (F) and alpha smooth muscle actin (SMA) (I), were poorly expressed demonstrating the absence of these markers in the study phenotype. The overlapping of the expression of the markers corroborated the identity of these proteins (D, G, J). [file Image_1.tiff]

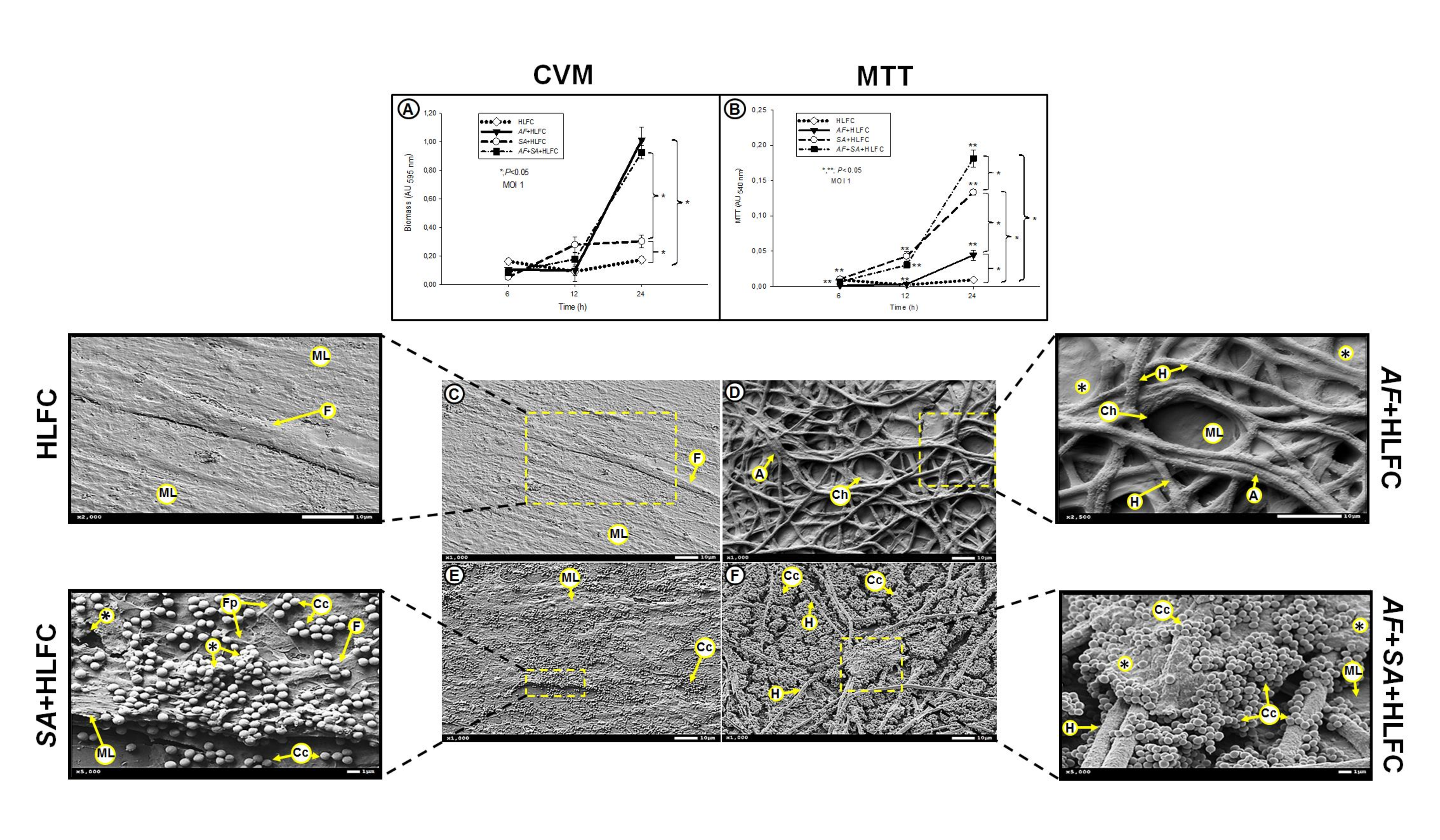

Supplement: Supplementary Figure 2 — Comparison of the analysis of the quantification and characterization methods of monoculture and mixed biofilms and MFBB on HLFC cultures. CVM) AF+HLFC and AF+SA+HLFC showed the highest biomass production (≈1.0 AU); followed by SA+HLFC (≈0.3 AU) which presented a lower biomass production (A: TTM) AF+SA+HLFC showed the most efficient metabolic activity (<0.20 AU); followed by SA+HLFC (<0.15 AU) and AF+HLFC (<0.05 AU) (B: SEM) The HLFC cultures infection free (C: 1000x; 2000x) were observed without apparent alteration (normal size). Observation of the AF+HLFC model showed that hyphae produced a ECM although with a worn-out appearance in some of them. The HLFCs were observed abnormal in size and shape (D: 1000x; 2500x). Model SA+HLFC exhibits a few microcolonies formation with EPS production. HLFCs have a normal size, with a presence of surface cracks and developed filopodia (E: 1000x; 5000x). The micrographs of the AF+SA+HLFC model reveal that the bacteria is exceeding its growth compared to fungus and fibroblasts. Moreover, the monolayer was limited to certain areas and the HLFCs were observed abnormal on several fields (F: 1000x; 5000x). The results are four replicates of three different experiments: n=12. Significance was determined using the Student-Newman-Keuls test, with multicomparison of procedures and are indicated as: (*), P<0.050. HLFCs, Human Limbo-Corneal Fibroblast cells; AF, Aspergillus fumigatus; SA, Staphylococcus aureus; H, hypha; F, Fibroblast; A, Anastomosis; Ch, Channels; Asterisk (*): Extracellular matrix; ML, Monolayer; Fp, Filopodia; Cc, Cocci. [file Image_2.tiff]

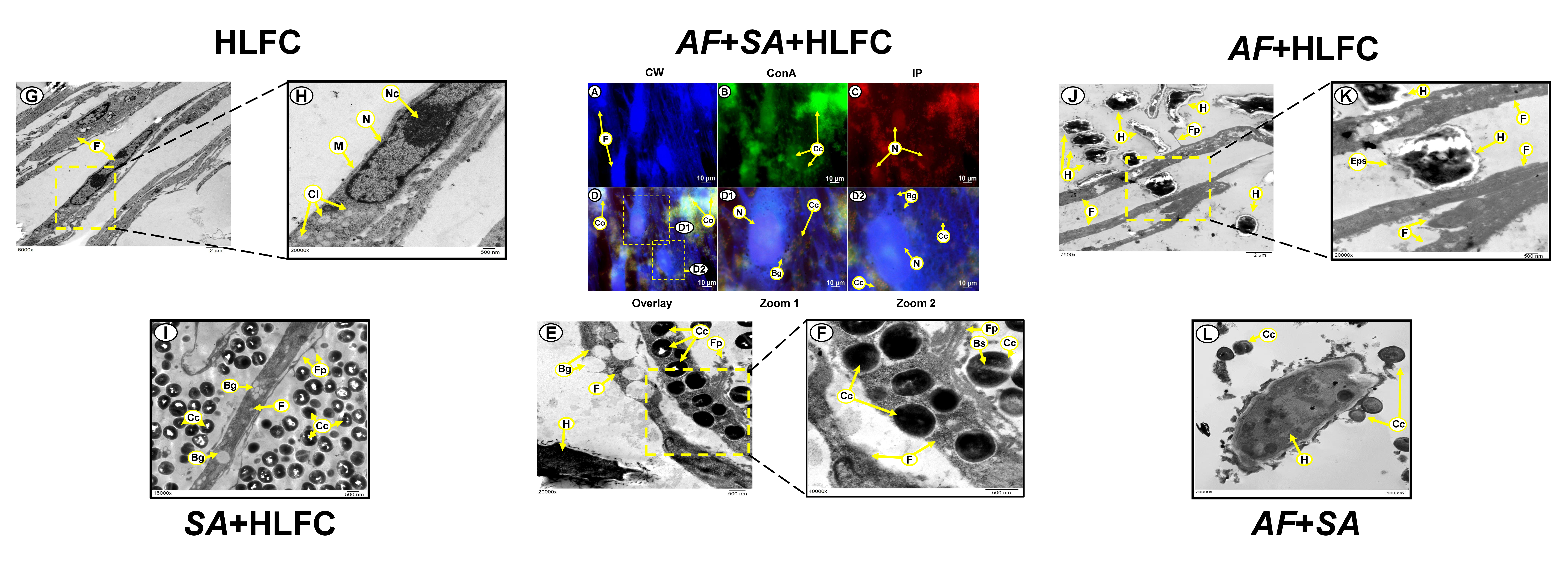

Supplement: Supplementary Figure 3 — Intracellular infection during FBI on HLFC. Biofilms were grown throughout 24 h on in vitro monolayer cultures of HLFCs. EFM) Fibroblasts were detected with CW (A: 63x). Stained Bacteria Con A) enveloping fibroblasts (B: 63x). The IP (C: 63x) showed the nucleus of the HLFCs as red halos. The overlay of the images shows that the bacteria are distinguished as intense orange marks surrounding the HLFCs (D: 63x). A digital zoom showed interstitial of around 1 µm (D1; D2). TEM. AF+SA+HLFC: Fungal population was reduced compared to the cocci surrounding HLFCs (E: 20000x). In the cytoplasm of HLFCs intracellular cocci were observed; fibroblast formed interstitial (<1.0 µm) (F: 40000x). HLFC: Cells without infection were seen with their cytoplasm and internal structures unaltered. In addition, intracytoplasmic inclusions approximate size of 0.3-0.5 µm was observed (G: 6000x; H: 20000x). SA+HLFC: High bacterial population compare to fibroblasts. In the center of the micrograph, a HLFC with interstitial caused by intracellular infection of SA. (I: 15000x). AF+HLFC: Hyphae were seen secreting extracellular material between a group of fibroblasts (J: 7500x). In this model, HLFCs with interstitial were not observed; and some cellular structures can be observed within the cytoplasm (K: 20000x). AF+SA: During this FBI, it is possible to observe abnormal hyphae with polar invaginations, as well as adjacent cocci that attach to the fungal cell wall (L: 20000x). HLFC, Human Limbo-Corneal Fibroblasts; AF, Aspergillus fumigatus; SA, Staphylococcus aureus; F, Fibroblast; H, Hypha; Cc, Cocci; Co, Co-localization; Bg, Interstitial; Fp, Filopodia; Eps, Exopolymeric substance; Bs, Bacterial septum; M, Cytoplasmic membrane; N, Nucleus; Nc, Nucleolus; Ci, Cytoplasmic inclusions; Mv, Microvesicles. [file Image_3.tiff]

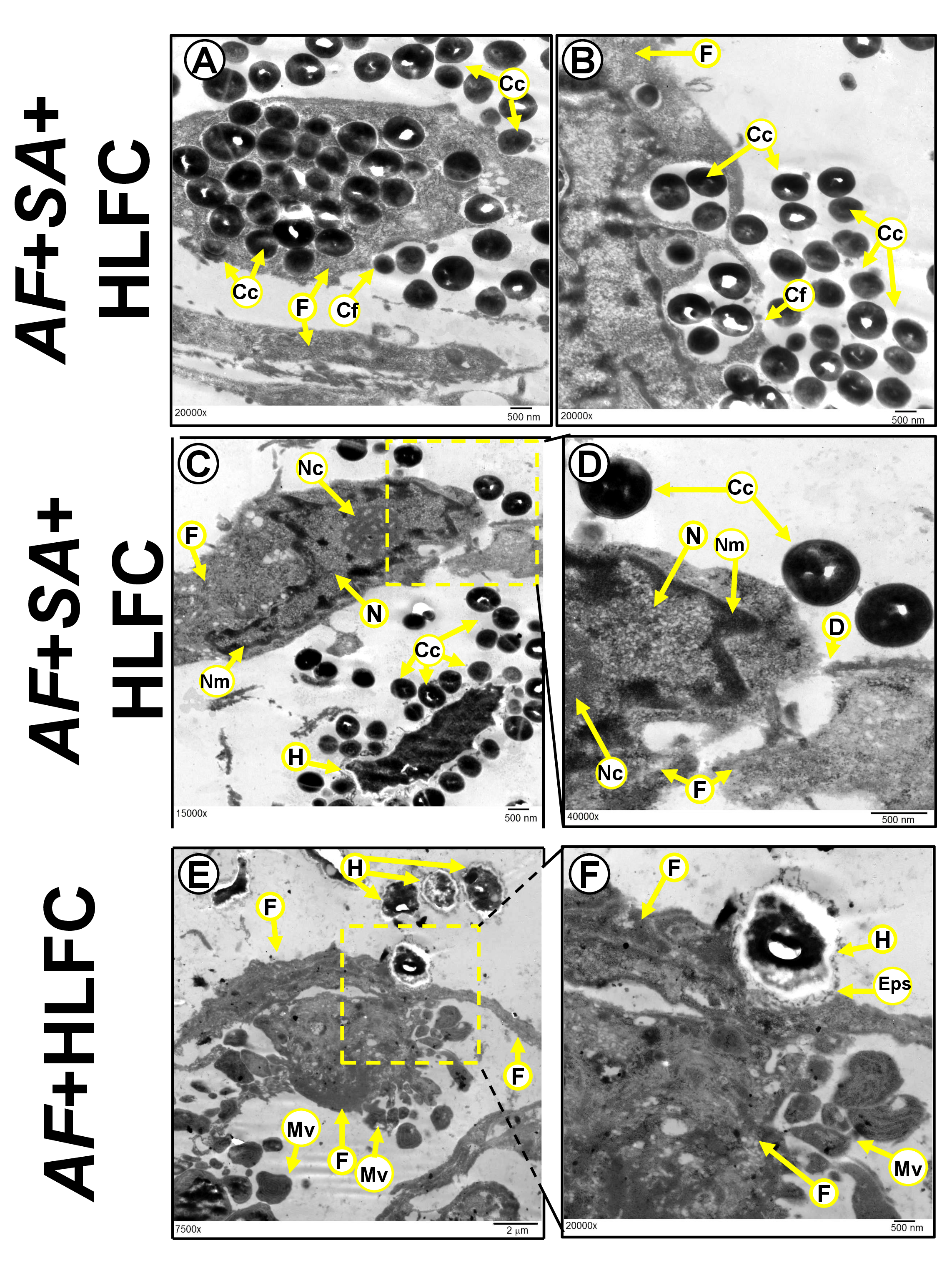

Supplement: Supplementary Figure 4 — Behavior of HLFC during FBI observed by TEM at 24 h. Intracellular cocci dividing (presence of bacterial septum) a crescent formation is distinguished (A: 20000x; B: 20000x). Cocci surrounded deformed hyphae (C: 15000x). Zooming permitted to observe desmosomes (D: 40000x). AF infection caused heterogeneous cytoplasm and adjacent microvesicles to the cytoplasmic membrane of HLFC (E: 7500x; F: 20000x); hyphae perforate the cytoplasmic membrane. HLFC, Human Limbo-Corneal Fibroblasts; AF, Aspergillus fumigatus; SA, Staphylococcus aureus; H, hypha; F, Fibroblast; M, Cytoplasmic membrane; Nm, Nuclear membrane; N, Nucleus; Nc, Nucleolus; Eps, Exopolymeric substance; Cc, Cocci; Bg, Interstitial; Bs, Bacterial septum; Mv, Microvesicles; Cf, Crescent formation; D, Desmosomes. [file Image_4.tiff]
